# Application of Cold Wire Gas Metal Arc Welding for Narrow Gap Welding (NGW) of High Strength Low Alloy Steel

**DOI:** 10.3390/ma12030335

**Published:** 2019-01-22

**Authors:** Rafael A. Ribeiro, Paulo D. C. Assunção, Emanuel B. F. Dos Santos, Ademir A. C. Filho, Eduardo M. Braga, Adrian P. Gerlich

**Affiliations:** 1 Centre for Advanced Materials Joining (CAMJ), University of Waterloo, 200 University Avenue West, Waterloo, ON N2L 3G5, Canada; agerlich@uwaterloo.ca; 2 Metallic Materials Characterization Laboratory (LCAM) Federal University of Pará (UFPA), Rua Augusto Corrêa, 1—Guamá, Belém, PA 66075-110, Brazil; pd2costa@uwaterloo.ca (P.D.C.A.); eng.angelo80@gmail.com (A.A.C.F.); edbraga@ufpa.br (E.M.B.); 3 Liburdi Automation Inc., Liburdi GAPCO, 400 ON-6, Dundas, ON L9H 7K4, Canada; esantos@liburdi.ca

**Keywords:** GMAW, CW-GMAW, Narrow gap welding, sidewall penetration, high strength steel, X80

## Abstract

Narrow gap welding is a prevalent technique used to decrease the volume of molten metal and heat required to fill a joint. Consequently, deleterious effects such as distortion and residual stresses may be reduced. One of the fields where narrow groove welding is most employed is pipeline welding where misalignment, productivity and mechanical properties are critical to a successful final assemblage of pipes. This work reports the feasibility of joining pipe sections with 4 mm-wide narrow gaps machined from API X80 linepipe using cold wire gas metal arc welding. Joints were manufactured using the standard gas metal arc welding and the cold wire gas metal arc welding processes, where high speed imaging, and voltage and current monitoring were used to study the arc dynamic features. Standard metallographic procedures were used to study sidewall penetration, and the evolution of the heat affected zone during welding. It was found that cold wire injection stabilizes the arc wandering, decreasing sidewall penetration while almost doubling deposition. However, this also decreases penetration, and incomplete penetration was found in the cold wire specimens as a drawback. However, adjusting the groove geometry or changing the welding parameters would resolve this penetration issue.

## 1. Introduction

Narrow gap welding (NGW) is a technique used to weld thick joints with the aim of reducing the molten metal deposited volume, ultimately decreasing distortion and residual stresses caused by thermal stresses developed during welding. One of the drawbacks associated with this technique is sidewall penetration caused by the arc wandering during welding. This problem is so critical in heavy duty welding that models to predict it are found in the literature [1].

Various NGW variants were developed to overcome such drawbacks: rotating arc, swing arc and wave-shaped wire systems. Another reason to use these modified systems in narrow gap welding is to improve the wettability of the sidewall by the welding pool and to better distribute the heat across the weld to mitigate distortions. A common setback for these systems is their expense and the use of highly trained personnel in their operation, which considerably increases the costs of manufacturing.

Not only are arc-modified processes employed in NGW, but also increased-deposition processed are successfully used, e.g., tandem gas metal arc welding (T-GMAW) [2] and twin GMAW [3]. However, these processes rely on increased deposition, while they increase the amount of heat transferred to the substrate, which might cause increased distortion or residual stresses. To mitigate distortion and other heat-induced detrimental effects, cold metal transfer (CMT) was developed, in which the wire feed motion is reversed at a time synchronized with the pulse current to optimize the metal transfer. A review of the applications of CMT emphasizing the low dilution and the effect of post weld heat treatments (PWHT) in various materials can be found in [4].

One alternative to these sophisticated welding processes is the cold wire gas metal arc welding (CW-GMAW), which consists of the standard gas metal arc welding (GMAW) with an extra cold wire (non-energized) fed into the arc-welding pool system. This process increases the deposition of the welds while maintaining the nominal heat input for the same welding parameters in standard GMAW. Previous research [5] has shown that the feeding of cold wire causes a slight increase in current without a corresponding increase in penetration. A feature that distinguishes GMAW and CW-GMAW is the reduced dilution caused by cold wire feed rates, which decreases penetration and melting of the base metal. Ultimately, the two processes differ by range of admissible parameters, since the cold wire can stabilize metal transfer [6]. Moreover, this difference in dilution can be linked to the thermal signature, as reported in [7], showing that cold wire welds result in lower heat-induced distortion compared to standard GMAW welds.

Previous research [8] demonstrates the possibility of welding U-grooves using CW-GMAW with pure carbon dioxide as shielding gas, and this method was primarily developed to be applied in shipbuilding. The feasibility of this process to weld a 5 mm wide groove was recently studied on ASTM A131 grade A steel, revealing that, due to the pinning of the arc to the cold wire, the sidewall erosion was considerably reduced in comparison to welds manufactured with standard GMAW [9]. Subsequent research [10] studied the effect of the CW-GMAW on process-induced residual stresses, concluding that welds manufactured by CW-GMAW have lower levels of residual stresses. This decrease in residual stresses might explain the improvement in fatigue life reported in [11].

The present work reports the further development on the work of prior research [9], and reports preliminary results regarding the application of CW-GMAW to weld high strength pipeline steels in narrow gap configuration. The feasibility of NGW employing CW-GMAW was assessed by comparing the severity of sidewall erosion in the welds and the presence of defects in the root pass. The results point to the general feasibility of NGW using CW-GMAW pipeline applications, and illuminate possible future work to avoid certain defects found during welding. The arc attachment to sidewalls can be ascribed to the arc shortest electron path according to Zhang et al. [12].

## 2. Experimental Set-Up and Materials

**Figure 1:**
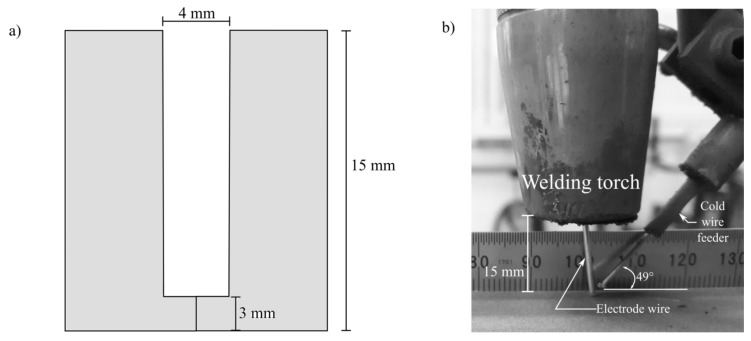
Schematics of the groove geometries used in this work: (**a**) schematic of the narrow groove, showing the cross-section; and (**b**) detail of CW positioning and feeding angle.

NGW were fabricated using both GMAW and the cold wire gas metal arc welding (CW-GMAW) process. Figure 1 shows a schematic of the geometry of the narrow groove used in this work and the detail of cold wire positioning regarding the wire electrode and cold wire feeding angle. The grooves were welded using a Lincoln R500 welding power source linked to a Fanuc ArcMate 120i robotic arm. The size of the joints was 140 mm (length) × 115 mm (width) × 15 mm (thickness). Figure 1 shows groove gap and the root face. To determine the reproducibility, three replicates were manufactured for each welding condition. It is important to mention that the welds were performed in constant voltage mode, where no synergic controls were used during welding to adaptively control the arc dynamics.

To manufacture the welds, ER100S-G in the diameter of 1.2 mm was used as electrode, while the cold wire had a diameter of 1.0 mm. API X80 [9] was selected as base metal. The nominal compositions of the electrodes and of the base metal are given in Table 1. Moreover, no weaving or preheating was used during welding.

Cold wire feed rates are expressed here as a fraction of the electrode mass feed rate, due to the fact that two wires of different diameters were used for the electrode and cold wire, respectively. To quantify the cold wire feed rate as function of the electrode wire, the mass percentage as a fraction of the electrode was used. The mass feed rate was calculated from the wire density and cross-sectional area.

To evaluate the welding process, a NGW joint design used in heavy welding applications such as pipeline welding was considered. This configuration was chosen to demonstrate an immediate application of the process, which is critical to welding of thick structures, since it decreases the heat-induced detrimental effects of welding passes.

During welding, the current and voltage signals were acquired at the sampling frequency of 20 kHz for 2 s with synchronized high speed imaging at 5000 fps with shutter speed of 25 ms, an aperture of f/22, and a narrow band pass filter of 900 ± 10 nm. The videography was performed with the camera in parallel to the groove longitudinal line to record the metal transfer inside the groove. The high speed images shown in this work were selected to adequately represent the arc dynamics and metal transfer, when in a stable condition. The welding parameters used are reported in Table 2. For all welds, the shielding gas mixture used was Ar-15%CO${}_{2}$ at a flow rate of 17 L/min, and the contact tip to work-piece distance (CTWD) was constant and equal to 17 mm. The welding parameters were set to apply the same heat input using both processes. The quantities of average voltage ($U_{average}$), average current ($I_{average}$), and average power ($P_{average}$) were calculated using the average instantaneous algorithm according to Equations (1)–(3):

(1) $$U_{average}=\sum_{1}^{i}U_{i}$$

(2) $$I_{average}=\sum_{1}^{i}I_{i}$$

(3) $$P_{average}=\sum_{1}^{i}U_{i}\times I_{i}$$

The arc arc stability was also assessed through cyclogrammes which are voltage versus current plots, and are used to study the events occurring in the arc electric. They are useful since they show the amount of short-circuits and account for the general arc stability of the process [13]. For a more thorough discussion on cyclogrammes and their respective zones, the reader should refer to prior work [14].

Once the experiments were completed, the specimens were subjected to standard metallographic procedures and etched with 5% Nital to reveal the macrostructure. The cross-sections showing the passes sequence were taken from the start, middle, and end of the joints. The cross-sections showing the complete joint were taken from the middle of the joints. The hardness map was performed with a 200 g load and 10 s dwell time. The distance between indentations was 0.3 mm and the distance between lines was 0.3 mm.

## 3. Results

### 3.1. Electrical Data

Table 3 presents the actual electrical data for current, voltage and power probed for the welding conditions employed in this work. For GMAW and CW-GMAW, the heat input per pass was similar.

#### 3.1.1. Oscillograms

**Figure 2:**
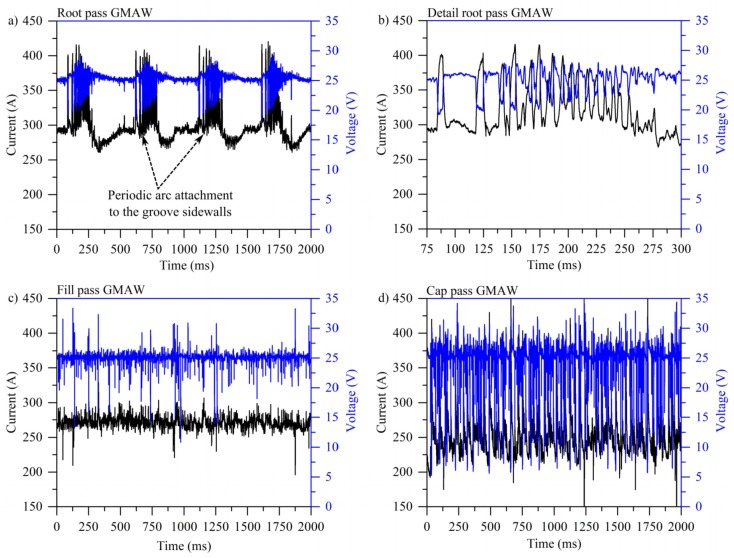
Typical oscillograms for the GMAW condition: (**a**) root pass; (**b**) detail of the root inside the period of repetition; (**c**) fill pass; and (**d**) cap pass.

**Figure 3:**
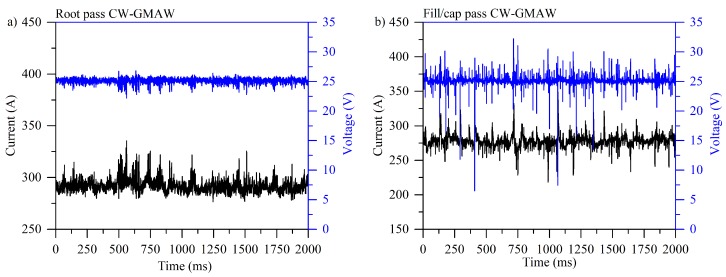
Typical oscillograms for the CW-GMAW condition: (**a**) root pass; and (**b**) fill/cap pass.

Figure 2 shows the oscillograms for the standard GMAW condition. For the root pass, one can notice a periodic repetition with frequency of approximately 3 Hz (Figure 3a). By correlating the electrical signals to the high speed images, it was observed that this repetition is a consequence of arc attachment to the groove sidewalls. As a consequence of the arc attachment to the sidewall, the power source promotes an instantaneous increase in the arc current, as shown in detail in Figure 3b. It is possible that this was caused by the auto-regulation system of the power source, which interprets the attachment of the arc to sidewalls as an increase in wire feed speed, consequently increasing the current to keep the melting rate constant while the arc length is reduced due to the arc attachment to the groove walls.

On the other hand, one can notice that this repetitive behavior did not occur in the fill and cap passes (Figure 2c,d), likely due to a reduced degree of arc constriction, and the shortest path to the electron conduction being the bottom of the groove and not the sidewalls. On the other hand, the severity of short-circuits in the cap pass was higher when compared to the filler pass. This suggests that the distance between the droplet (as shown in the high speed frames, Figure 6) and the substrate is lower, favoring short-circuits.

Figure 3 shows the oscillograms for the CW-GMAW specimens. It is noted that the periodic pattern observed in the oscillograms of GMAW for root pass was not observed for the entire sampling period in CW-GMAW of 2000 ms. This suggests the sidewall erosion was mitigated during CW-GMAW, since sidewall penetration causes the periodicity in the observed signal. Moreover, the short-circuit severity in the fill/cap pass is less prominent than that observed in the GMAW specimen.

#### 3.1.2. Cyclogrammes

**Figure 4:**
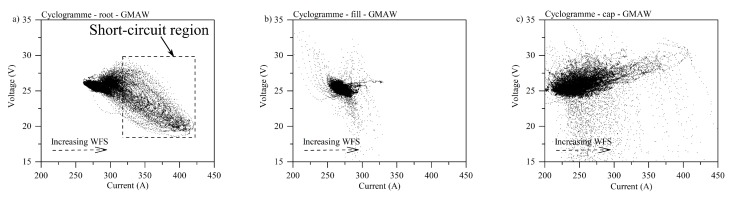
Cyclogrammes for the GMAW specimens: (**a**) root pass; (**b**) fill pass; and (**c**) fill pass.

Figure 4 shows typical cyclogrammes for GMAW specimens where one can notice that in the root pass (Figure 4a) the number events corresponding to short-circuits (region inside the dashed rectangle) are larger than the arc burning area (darker region, upper left-side of the dashed square). Ultimately, this cyclogramme points out an unstable condition where the arc burning region is smaller compared to the perturbed region.

As reflected by oscillograms, the fill pass was more stable, with some short-circuits and high voltage points, indicating large variations in arc length (see Figure 4b). In the cap pass (Figure 4c), one can notice a larger short-circuit region with slight variation in arc length. The results also suggest that there was a high variation in current, probably due to an increased quantity of metal to melt, interpreted by the power source as an increase in wire feed speed (higher melting speed).

**Figure 5:**
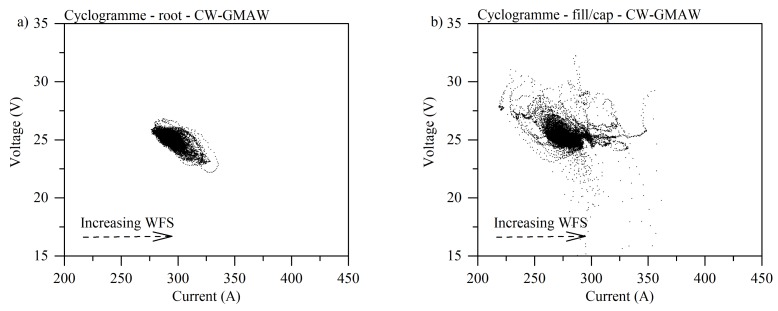
Cyclogrammes for the GMAW specimens: (**a**) root pass; (**b**) fill pass; and (**c**) fill pass.

Figure 5 shows the cyclogrammes for the CW-GMAW condition. One observes that, by comparing the cyclogrammes for root pass between standard GMAW and CW-GMAW, the arc burning operation range for CW-GMAW is shorter, indicating that this was more stable than the root in GMAW condition. One observes the complete absence of short-circuits (see Figure 5a). The cause of such stabilization should be attributed to the cold wire feed. Meanwhile, comparing the fill/cap condition (Figure 5b) between CW-GMAW and GMAW, one finds short-circuits events where voltage dropped below 20 V, along with variations in arc length, suggested by high values of voltage for the same range of current.

### 3.2. High Speed Imaging

**Figure 6:**
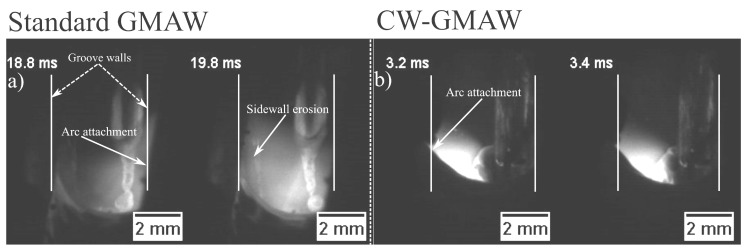
High speed frames, arc dynamic behavior inside the groove: (**a**) standard GMAW; and (**b**) CW-GMAW.

Figure 6 shows the high speed frames for the standard GMAW and CW-GMAW inside the grooves. As suggested by the electrical signals, it was possible to verify that the arc for the GMAW often attaches to the sidewalls, thus eroding it. The same author claims that the arc attachment was avoided when welding in constant current mode, since, in these sources, to maintain and almost constant melting speed, the voltage highly varies. Meanwhile, in CW-GMAW, the arc attachment was to the cold wire, which prevents sidewall erosion. In Figure 6a, one can discern that the arc is pinned to the rear (cold wire) and not to the sidewalls.

### 3.3. Sidewall Penetration

The erosion of the sidewall was caused by the arc self regulation dynamics, which tried to establish the shortest path to electron flow, thereby increasing the melting rate. However, as the arc moved through the groove, the attachment point was continually changing, melting multiple points across the groove walls. This multiple erosion points might have detrimental effects on the mechanical properties of the joint.

**Figure 7:**
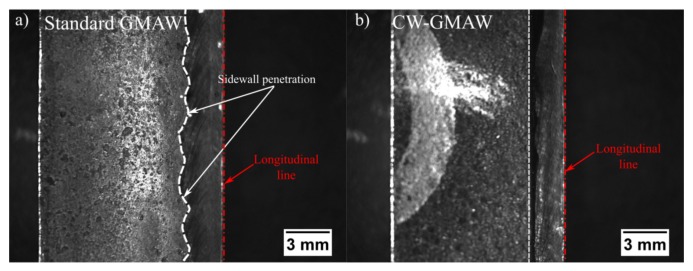
Top view of the sidewall penetration caused by the root pass; the specimens were cut along the longitudinal line (dashed red line): (**a**) standard GMAW; and (**b**) CW-GMAW. Once can note that the specimens were cut along the longitudinal axis of the joint. Note: In (**b**), the mark on its front is a written identification of the sample that could not be removed.

Figure 7 shows the sidewall penetration caused by the root pass during welding. One observes that the standard GMAW erodes the sidewalls consistently with the oscillograms shown in Figure 2a. There, every time the arc attached to the sidewalls, intense and fast short-circuiting caused the current to increase abruptly (to fuse the extra metal and restore the compatible voltage (arc length, in constant voltage) to the current pre-set before welding). Conversely, as in the CW-GMAW, the arc was attached to the cold wire, thus there is effectively no sidewall penetration, as can be seen in Figure 6a.

### 3.4. Evolution of the HAZ in Root Pass

**Figure 8:**
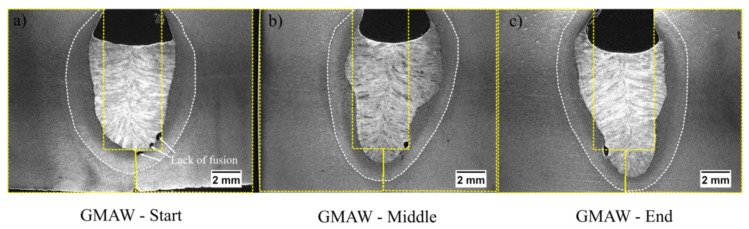
Root pass macrographs for the standard GMAW process: (**a**) start of the joint; (**b**) middle of the joint; and (**c**) end of the joint.

Figure 8 shows the evolution of the heat affected zone (HAZ) in the root pass for the standard GMAW welds in three different locations of the bead: start, middle and end. One can find that the arc wandering resulted in welds that are rather non-symmetrical. This was likely due to the inconsistency in penetration, associated with is incomplete fusion defect. Moreover, the size of the root face also contributed to this issue. In addition, one can note in Figure 8 that, as the weld progressed, the plate became hotter and consequently penetration was slightly increased in the end of the weld.

**Figure 9:**
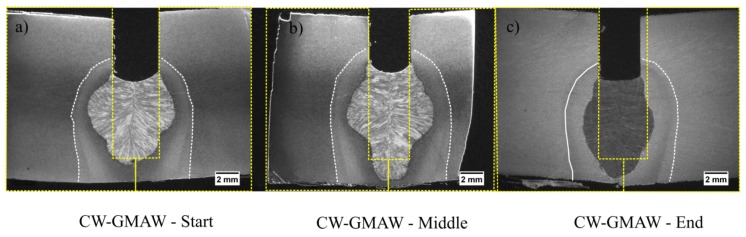
Root pass macrographs for the CW-GMAW process: (**a**) start of the joint; and (**b**) middle of the joint; and (**c**) end of the joint.

Figure 9 shows the root pass in three locations across the bead for CW-GMAW; no discontinuities (based on cross-sections inspection) were likely to occur in the root pass, and, generally, the sidewall penetration in the root was much more symmetrical due to the arc pinning to the cold wire. One finds that the increase in deposition decreased the penetration of the weld, causing incomplete penetration.

### 3.5. Macrographs

**Figure 10:**
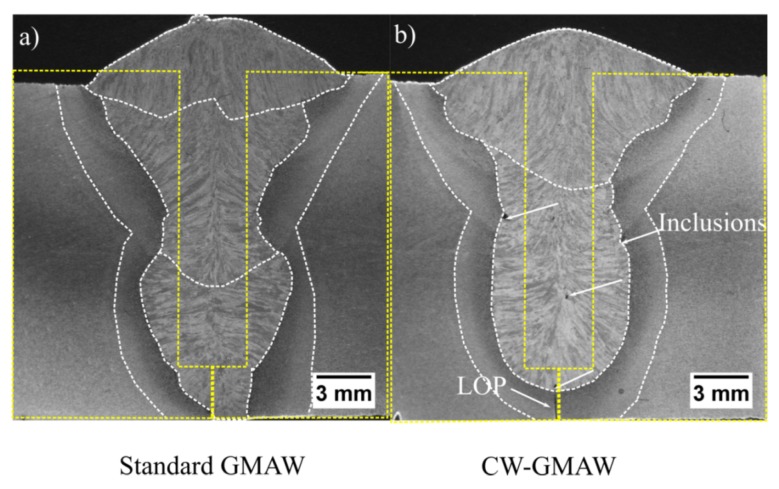
Middle of the bead cross-section showing the final weld morphology: (**a**) standard GMAW; and (**b**) CW-GMAW. The arrows indicate discontinuities such as inclusions and incomplete fusion.

Figure 10 shows cross-sections from the middle of the bead for both the standard GMAW and CW-GMAW. One notices that the middle cross-section of the standard GMAW has an acceptable morphology without discontinuities, while achieving suitable penetration in the root face. Conversely, incomplete penetration persists in CW-GMAW. Due to the cold wire feed rates, more mass was deposited in CW-GMAW, which caused a decrease in penetration. This accounts for the incomplete penetration. Another technicality in CW-GMAW is the presence of inclusions, most likely oxides, due to the increased level of titanium in the weld metal due to the cold wire feeding, as the same wire was used as electrode and cold wire.

The presence of inclusions points to the need for more careful grinding after the root to clean the silicates formed during welding pool solidification. Another feature that differs between the cross-sections is the quantity of passes to fill them out. Standard GMAW required three passes and CW-GMAW only two. Ultimately, the overall heat input in CW-GMAW was lower due this difference in number of passes.

To mitigate incomplete fusion, two alternatives might be used: increase the current by means of wire feed speed to increase penetration, or decrease the root face height to facilitate higher penetration.

### 3.6. Micrographs

The HAZ width of the standard GMAW and CW-GMAW specimens were compared. The intercritical heat affected zone (ICHAZ), which is basically formed between 800 and 500 ${}^{\circ }$C upon cooling, is narrower in CW-GMAW welds compared to GMAW.

#### Weld Interface—Root Pass

**Figure 11:**
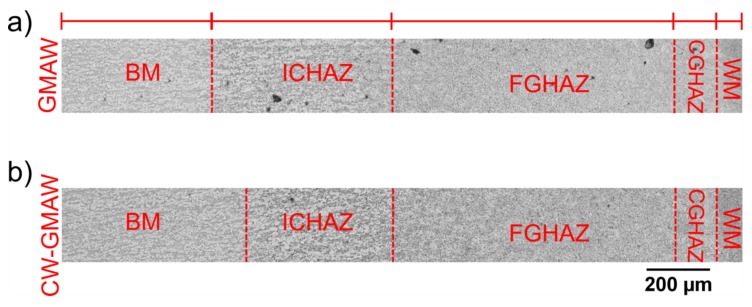
Heat affected zone (HAZ) width in the GMAW specimen showing the Weld metal (WM), Coarse grain heat affected zone (CGHAZ), Fine grain heat affected zone (FGHAZ), Intercritical heat affected zone (ICHAZ), and Base metal (BM): (**a**) standard GMAW; and (**b**) CW-GMAW.

Figure 11a shows the HAZ from the weld interface to base metal in the conventional GMAW specimen. Figure 11b shows the HAZ from the interface in the CW-GMAW specimen. In both specimens, the images were taken at the root pass, in a one pass weld (see Figure 8b and Figure 9b, respectively).

One can see that the ICHAZ is slightly narrower in CW-GMAW than in the standard GMAW. This is consistent with previous research. This seems to indicate that CW-GMAW specimens had a shorter cooling time between 800 and 500 ${}^{\circ }$C compared to the conventional GMAW specimens. This seems to indicate that less heat was actually applied across the weld joint.

### 3.7. Vickers Hardness

**Figure 12:**
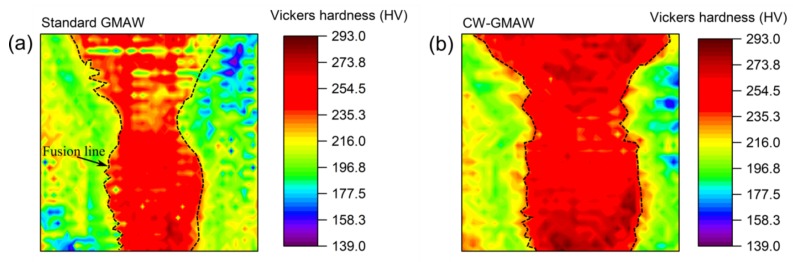
Hardness map of the cross-sections: (**a**) standard GMAW; and (**b**) CW-GMAW, with weld interface marked by dashed lines.

To characterize the strength of the of the joint via cross-sections, maps of the Vickers hardness values are reported here. Figure 12 shows the hardness map of the completed joint for standard GMAW and CW-GMAW specimens.

One can see that the Vickers hardness in the weld for the CW-GMAW is higher, which indicates that the cooling rate of the weld metal in CW-GMAW was faster than the standard GMAW. For some conditions, the melting efficiency of CW-GMAW was higher than in conventional GMAW. The melting efficiency is the amount of heat that actually transfers to the melting pool over the welding total power, and the difference in this value might explain slower cooling rates.

**Figure 13:**
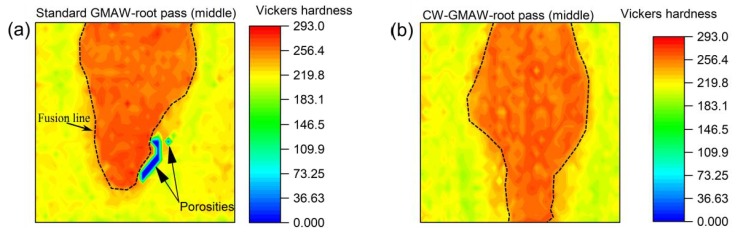
Hardness map of the root pass, middle cross-section: (**a**) standard GMAW; and (**b**) CW-GMAW, with weld interface marked by dashed lines.

Figure 13 shows the hardness map of the root pass in cross-section extracted from the middle of the joint. Regarding the root pass, one can see that the values are similar in standard GMAW and CW-GMAW. However, comparing Figure 12 and Figure 13, one can see that the bottom of the CW-GMA welds is harder than the bottom of conventional GMAW. Given that the hardness patterns of the roots were similar, one has to consider that the cooling rate near the root was faster in CW-GMAW. However, the exact mechanism behind this fact is still unclear, but may be due to a faster heat transfer due to a larger contact area (the area of contact between the weld metal and the base metal) as well as a higher gradient between the weld and base metals in CW-GMA welds.

## 4. Discussions

The electrical oscillograms in Figure 2 show that the standard GMAW experiences some sort of oscillating pattern. It was noticed that this pattern corresponds to the instant the arc eroded the sidewalls. Moreover, the author pointed out that the arc attached to sidewall such that the current flows in the shortest path, thus the arc potential in the arc column remains at the minimum possible level.

This phenomenon in constant voltage (CV) leads ultimately to the melting of the contact tip and interruption of the welding processes. However, it was noted that, in constant current (CC), this process did not occur since the reduction in arc length, induced by the arc attachment to the sidewall, caused a small reduction in arc length in comparison the reduction in current, thus the power source reached a new equilibrium point.

On the contrary, in CW-GMA welding, the current path was shortest to the cold wire, which caused the arc to climb to it. This phenomenon prevented the arc attachment to the sidewalls, and consequently prevented sidewall erosion. Moreover, one observes that the arc was much more stable in CW-GMA welding (Figure 3) than in standard GMAW (Figure 2). The cyclogrammes for the the two cases confirmed this assertion. The root pass performed using CW-GMAW (Figure 5a) was more stable than in the conventional GMAW (Figure 4a), which had a tail that points to short-circuits caused by the sidewall erosion (dashed square).

Regarding the cap pass, one can observe that, in conventional GMAW, the arc tended to climb towards the contact, as indicated by the higher values of voltage (increase in arc length) and current in Figure 4c. However, in the CW-GMAW, such phenomenon did not occur as systematically, with only occasional values of current and voltage tending to the high voltage region. High speed images were used to investigate the arc dynamical behavior. The images show that the arc attached to the sidewalls in standard GMAW because of the internal regulation of the source in CV, as discussed above (Figure 6). In CW-GMAW, as expected from the electrical signals, no sidewall erosion was detected, as can be seen in Figure 6 and Figure 7.

Figure 8 shows the macros of the joint manufactured using conventional GMAW. One can observe that some fusion points are lacking. These were caused by the arc climbing causing over-melts in one direction, but leaving a gap and causing the lack of fusion. On the contrary, in CW-GMAW, one can notice that, as the arc is pinned to the cold wire, this causes a more stable melting pattern avoiding incomplete fusion across the joint (Figure 9).

The macros, taken from the middle of joints (Figure 10), show the difference in productivity. The standard GMAW joint was completely filled with three passes while the the CW-GMAW was filled with two passes. However, one observes that, in relation to the cold wire joint, there was a lack of penetration due to the arc pinning to the cold wire, which limits the penetration and dilution.

Figure 11 compares the HAZ for the two processes: standard GMAW and CW-GMAW. The difference in their size can be attributed to differences in thermal signature of CW-GMAW in comparison to GMAW. The difference in ICHAZ might be linked to higher thermal gradient in CW-GMAW than in conventional GMAW. This thermal gradient might result from an improved melting efficiency in CW-GMAW for some conditions.

Figure 12 and Figure 13 show the hardness maps over the complete macro and the root pass for both conventional GMAW and CW-GMAW, respectively. The Vickers hardness values in CW-GMAW (Figure 12) point to shorter cooling time (higher cooling rate), which might be linked to the higher thermal gradient caused by the possibly higher melting efficiency in CW-GMAW. This might explain the higher hardness in CW-GMAW compared to the standard GMAW. Regarding the root pass, the difference in hardness was likely due to the larger gradient formed by the larger joined area in CW-GMAW root. This led to higher cooling rates in the reheated root of the weld metal, with higher hardness.

## 5. Conclusions

Narrow groove welds in API X80 were fabricated using the standard GMAW and the CW-GMAW to assess the process feasibility using CW-GMAW for a joint with 4 mm gap. Taking into account the results discussed, the following conclusions can be drawn:

1. The welds using GMAW process presented serious sidewall penetration that might compromise their integrity.
2. The welds fabricated using CW-GMAW did not present sidewall erosion during root pass, conversely the increase in deposition compromised the penetration in the root region.
3. The amount of inclusions found in the the cold wire welds might be linked to the grade of the welding wires used.

## Figures and Tables

**Table 1:** Nominal chemical composition of the welding wires and the base metal.

	C	Mn	P	S	Ti	Mo	Ni	Cr	Fe
ER100S-G	0.08	1.25–1.80	0.20–0.55	0.01	0.10	0.25–0.55	1.20–2.10	1.25–1.80	Balance
API X80	0.22	1.85	0.025	0.015	0.06	-	-	-	Balance

**Table 2:** Welding parameters.

Welding Process	WFS (m/min)	Voltage (V)	Travel Speed (m/min)	CWFR (%)	Deposition (kg/h)
GMAW	7.62	25	0.41	-	4.11
CW-GMAW	7.62	25	0.41	80	7.39

**Table 3:** Average electrical parameters sampled during welding and nominal resulting heat input as response of the welding power source.

Welding Process	Pass	Average Voltage (V)	Average Current (A)	Average Power (W)	NHI (kJ/mm)
GMAW	root	25.13	299.69	7520.13	1.11
	fill	25.13	270.64	6801.29	1.00
	cap	25.12	253.27	6427.07	0.95
CW-GMAW	root	25.14	292.20	7344.18	1.08
	fill/cap	25.16	277.95	6991.18	1.03
